# Automatic Classification of Barefoot and Shod Populations Based on the Foot Metrics and Plantar Pressure Patterns

**DOI:** 10.3389/fbioe.2022.843204

**Published:** 2022-03-23

**Authors:** Liangliang Xiang, Yaodong Gu, Qichang Mei, Alan Wang, Vickie Shim, Justin Fernandez

**Affiliations:** ^1^ Faculty of Sports Science, Ningbo University, Ningbo, China; ^2^ Research Academy of Grand Health, Ningbo University, Ningbo, China; ^3^ Auckland Bioengineering Institute, The University of Auckland, Auckland, New Zealand; ^4^ Faculty of Medical and Health Sciences, The University of Auckland, Auckland, New Zealand; ^5^ Department of Engineering Science, The University of Auckland, Auckland, New Zealand

**Keywords:** gait, barefoot, plantar pressure, foot shape, support vector machine (SVM), naive Bayes

## Abstract

The human being’s locomotion under the barefoot condition enables normal foot function and lower limb biomechanical performance from a biological evolution perspective. No study has demonstrated the specific differences between habitually barefoot and shod cohorts based on foot morphology and dynamic plantar pressure during walking and running. The present study aimed to assess and classify foot metrics and dynamic plantar pressure patterns of barefoot and shod people via machine learning algorithms. One hundred and forty-six age-matched barefoot (*n* = 78) and shod (*n* = 68) participants were recruited for this study. Gaussian Naïve Bayes were selected to identify foot morphology differences between unshod and shod cohorts. The support vector machine (SVM) classifiers based on the principal component analysis (PCA) feature extraction and recursive feature elimination (RFE) feature selection methods were utilized to separate and classify the barefoot and shod populations via walking and running plantar pressure parameters. Peak pressure in the M1-M5 regions during running was significantly higher for the shod participants, increasing 34.8, 37.3, 29.2, 31.7, and 40.1%, respectively. The test accuracy of the Gaussian Naïve Bayes model achieved an accuracy of 93%. The mean 10-fold cross-validation scores were 0.98 and 0.96 for the RFE- and PCA-based SVM models, and both feature extract-based and feature select-based SVM models achieved an accuracy of 95%. The foot shape, especially the forefoot region, was shown to be a valuable classifier of shod and unshod groups. Dynamic pressure patterns during running contribute most to the identification of the two cohorts, especially the forefoot region.

## 1 Introduction

The human being’s locomotion under the barefoot condition enables normal foot function and lower limb biomechanical performance from a biological evolution perspective (D’Août et al., 2009; Lieberman, 2012). Previous studies have found that foot morphology is different for the habitual shod and barefoot cohorts (D’Août et al., 2009; Shu et al., 2015). Functional performances of lower limbs during gait are affected by foot morphology (Zhang and Lu, 2020; Xiang et al., 2020a; Xiang et al., 2020b). Lieberman et al. (2010) demonstrated that barefoot runners with forefoot strike could decrease impact force compared to shod runners with the rearfoot strike pattern. Foot intrinsic muscle and longitudinal arch function may be affected for the habitually shod population (Lieberman, 2012; Davis et al., 2017). The barefoot population also presented a lower injury incidence in the ankle and knee joints than their shod counterparts (Altman and Davis, 2016). In a given year, 79% of shod runners suffered from running-related injuries (van Gent et al., 2007).

On the other hand, opponents argue that without the protection and cushioning function provided by modern shoes, the injury incidence of the foot and calf will be increased (Altman and Davis, 2016). Cushioning shoes have been highly researched in recent years (Sinclair et al., 2016; Chan et al., 2018; Hannigan and Pollard, 2019). Both instantaneous loading rate and peak tibial acceleration were significantly increased in barefoot running, providing no footwear midsole support, and cushioning, compared with cushioned shoes (Sinclair et al., 2016; Agresta et al., 2018). Barefoot running has gained popularity in recent years. However, runners transitioning to barefoot running are more prone to injury than the habitual barefoot population (Altman and Davis, 2016). Therefore, understanding foot biomechanical differences between barefoot and shod folks could help to decrease the injury rate among novice barefoot runners.

Plantar pressure is the salient parameter in gait evaluation and injury detection (Fernández-Seguín et al., 2014; Maiwald et al., 2018). Bergstra et al. (2015) found that running with minimalist running shoes increased the plantar pressure in the forefoot region compared to conventional running shoes. Given the gait differences cause by the shod habit, there is still a lack of understanding of the unique gait patterns of barefoot and shod people and how these differences are attributed to gait function performance and injury prevention.

Machine learning algorithms are widely used in sport-specific movement recognition and gait biomechanics (Halilaj et al., 2018; Cust et al., 2019). It can successfully identify and classify gait characteristics based on plantar pressure variables (Bennetts et al., 2013; Li et al., 2020). Naïve Bayes classifiers greatly simplify learning based on Bayes’ rule and assuming that the attributes are conditionally independent given the class (Rish, 2001). Physical activity and falls could be detected using wireless sensors embedded with the Naïve Bayes algorithm (Yang et al., 2010). The support vector machine (SVM) found the optimal separating hyperplane that maximizes the margin of separation between categories through a decision boundary (Vapnik, 1998). In SVM, the input matrix was transformed into a high dimension space using the different types of kernel algorithms, including linear and non-linear methods (Fukuchi et al., 2011). Wu and Wang (2008) reported that the SVM algorithms combined with principal component analysis (PCA)-based feature extract techniques could be used to classify gait patterns based on ground reaction force. Spatiotemporal features depicted good performance in identifying young and elderly gait pattern differences via SVM classifiers with a linear kernel (Taylor et al., 2013). Based on foot-ankle kinematics and kinetics data, SVM can classify runners with different running experience levels (Suda et al., 2020). The study from Clermont et al. (2017) separated and classified the competitive and recreational runners via the SVM model using lower limb kinematics as input data.

To the best of our knowledge, even though the present evidence illustrates the differences between habitually barefoot and shod groups, no study has demonstrated the specific differences between them based on foot morphology, and dynamic plantar pressure during walking and running. Traditional questionnaires on shoe-wearing habits are subjective and cannot provide objective foot shape and functional information. In contrast, machine learning with population-based foot shape and plantar pressure measures can be used to classify habitually barefoot and shod groups more broadly from an adapted biomechanics perspective. The primary objective of this study was to assess and classify foot shape and dynamic plantar pressure patterns between barefoot and shod populations via the Naïve Bayes and SVM algorithms. It was hypothesized that shod and unshod cohorts could be separated, and the differences between groups would be primarily related to the plantar pressure beneath the big toe and first-fifth metatarsals (M1-M5).

## 2 Materials and Methods

### 2.1 Participants

One hundred and forty-six age-matched barefoot (*n* = 78) and shod (*n* = 68) participants were recruited for this study. The anthropometric parameters included age: 21.3 years, mass: 68.7 ± 6.3 kg, height: 1.73 ± 0.06 m and BMI: 23.0 ± 1.3 kg/m^2^ for the barefoot population and age: 22.1 years, mass: 71.2 ± 6.1 kg, height: 1.76 ± 0.04 m and BMI: 22.9 ± 1.4 kg/m^2^ for the habitual shod population. The BMI of all included participants was within the normal range (BMI 18.5–25 kg/m^2^). The experimental and data collection protocol was approved by the local Ethics Committee (RAGH20170306). The unshod population was from southern Indian volunteers exhibiting habitual barefoot gait since birth, and the shod cohort was from China. All participants were free from lower limb injury in the previous 6 months and were informed of the experimental protocols, objectives, and requirements, and informed written consent was obtained from participants before the experiment.

### 2.2 Data Collection and Processing

**FIGURE 1:**
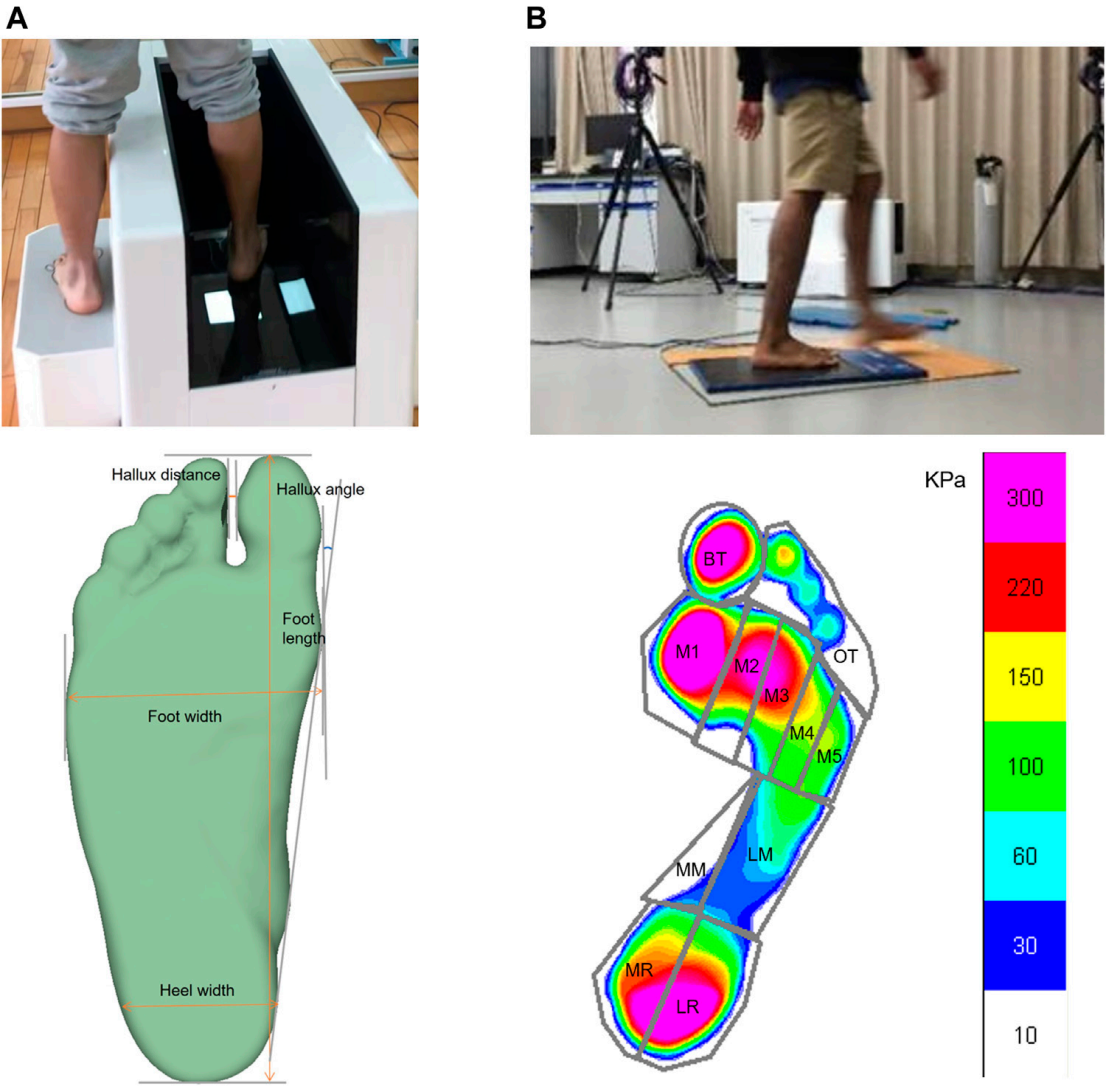
Foot morphology measurement and parameters **(A)** and Foot pressure measurement and plantar region division **(B)**.

the foot shape of each participants’ right foot was scanned using the Easy-Foot-Scan (OrthoBaltic, Kaunas, and Lithuania). The resolution, smooth factor, and hole filling parameters were set as 1.0, 30, and 100 mm, respectively (Xiang et al., 2018). Participants normally stand with shoulder width between their legs while scanning (Figure 1A). The plantar pressure was collected from a Novel EMED® force plate (Novel GmbH, Munich, Germany) fixed in the middle of a 15 m gait path with the same surrounding dimensions in the gait laboratory (Figure 1B). The frequency of recording the dynamic plantar pressure pattern was 100 Hz. Before the data collection, each participant spent 5 mins on the lab setting familiarization. A self-selected gait speed was adopted for each participant during plantar pressure collection to enable the natural gait patterns. Walking and running speeds were 1.3 ± 0.3 m/s and 3.0 ± 0.4 m/s for barefoot people and 1.2 ± 0.2 m/s and 3.0 ± 0.4 m/s for shod participants. A mid-gait protocol was used for both walking and running sessions (Wearing et al., 1999). Specifically, the fourth step was captured for each trial, followed by four steps after striking the pressure plate. More details of this experimental protocol can be reviewed in our previous study (Mei et al., 2019). Data were discarded if the participant presented any gait adjustment or the foot was not in full contact with the force plate for each trial. Finally, four successful trials with the right foot striking on the force plate of each session were obtained for further data processing. The mean of four trials for each participant was used for further analyses.

Foot morphology parameters included foot length, foot width, heel width, the distance between the hallux and the second toe (hallux distance), the angle between the hallux and the second toe (hallux angle), and arch index. Those six variables were inputted for the naïve Bayes classifier. The hallux angle is the angle created by the deviation of the hallux away from the tangent line connecting the medial heel and medial forefoot. Details of calculating the hallux distance and angle are shown in our previous study (Shu et al., 2015). We evaluated the arch index as the midfoot divided by the whole foot regions except the toes (Mei et al., 2019). With the assistance of the Novel data processing software (Munich, Germany), the pressure data were collected, including 11 regions within the foot plantar surface, specifically: big toe (BT), other toes (OT), M1-M5, medial midfoot (MM), lateral midfoot (LM), medial rearfoot (MR) and lateral rearfoot (LR), and with further details in our previous study (Mei et al., 2019). Peak pressure can well-represent foot loading characteristics during walking and running and is the most commonly used plantar pressure parameter in previous studies. Therefore, peak pressure was recorded in this study. So, 22 features were considered for the use of the SVM algorithm.

### 2.3 Data Preprocessing and Machine Learning Approaches

#### 2.3.1 Feature Extraction and Selection

**FIGURE 2:**
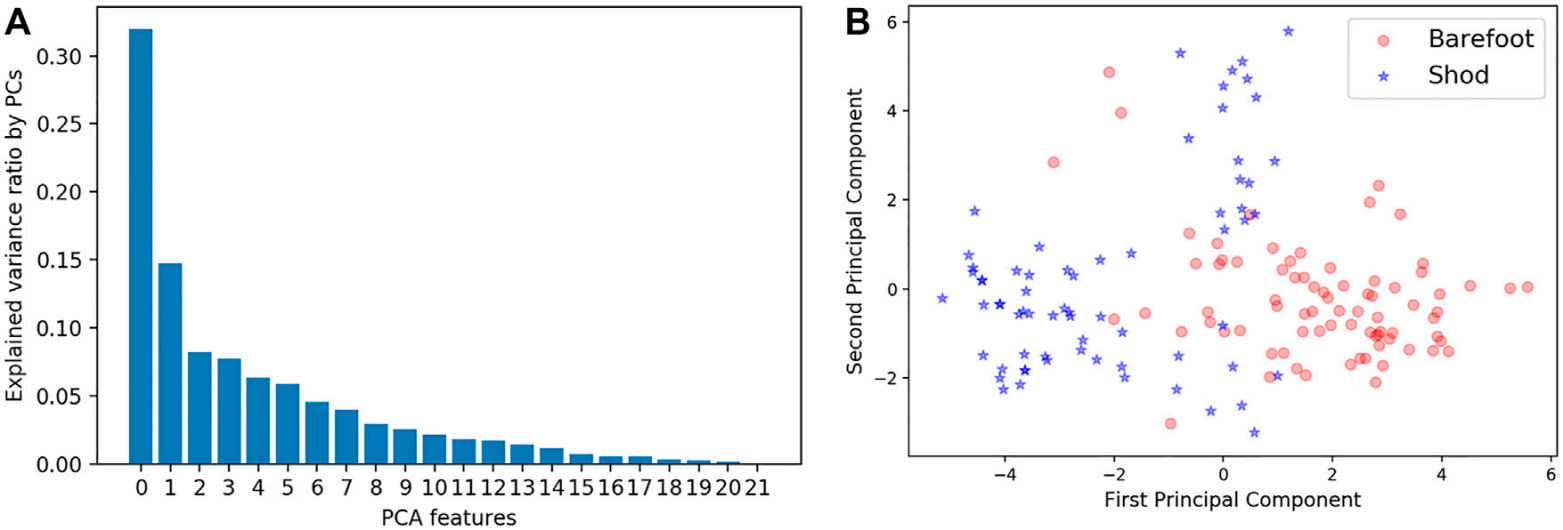
Explained variance percentage by each PC **(A)** and plot of 1^st^ and 2^nd^ PCs for barefoot and shod groups **(B)**.

The PCA algorithm was used for dimensionality reduction and feature extraction from raw plantar pressure data. The explained variance ratio for each principal component (PC) from 22 variables 1) and 1st and 2nd PCs classed for barefoot and shod groups 2) are depicted in Figure 2. The eigenvalues that explain the percentage of cumulative variance were set as 90%. Eleven PCs features were extracted for the SVM classifier.

**FIGURE 3:**
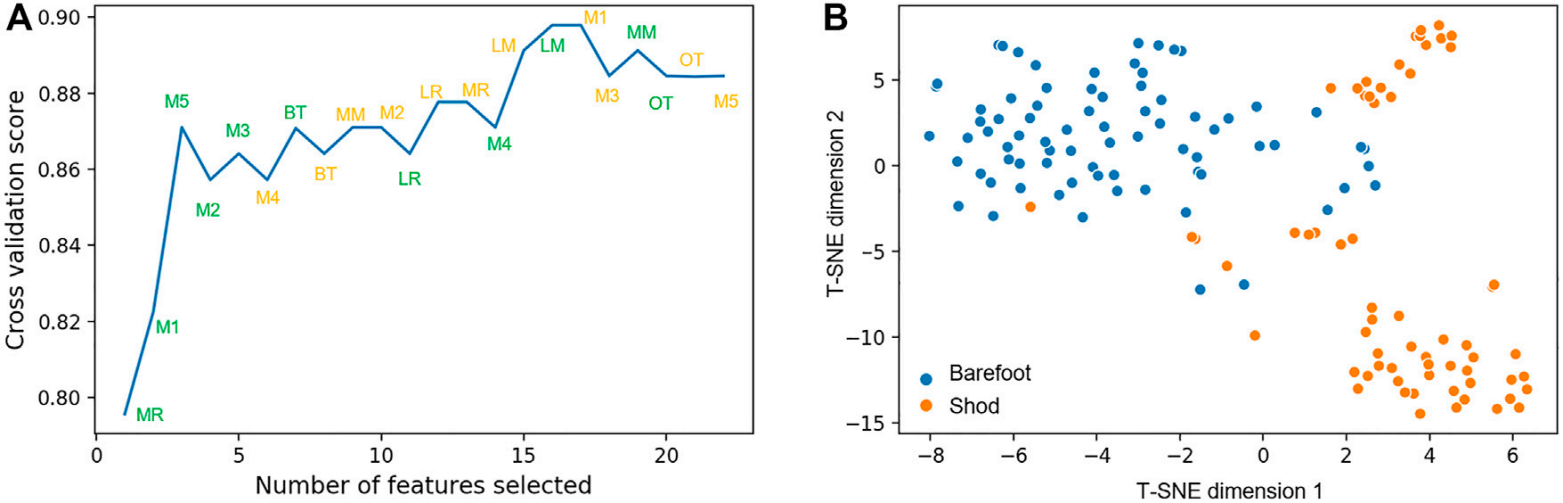
The number of features selected alter cross-validation score **(A)**, t-SNE visualization of selected 16 features **(B)**. Orange color denotes features from walking and green color indicates features from running.

A recursive feature elimination (RFE) method was used for variable selection. During the selection of the optimal number of features, SVC with a linear kernel was used as the estimator. To obtain an unbiased accuracy for the feature selection and keep the un-seen test data, the feature raking was integrated into a five-fold cross-validation procedure (Dindorf et al., 2021). After recursively ranking the features’ importance, 16 features were left preserving the highest cross-validation accuracy obtained from the whole 22 features (as shown in Figure 3A). These variables include BT, M2, M4, MM, LM, MR, and LR in walking plantar pressure pattern and BT, M1-M5, LM, MR, and LR in running gait. t-Distributed Stochastic Neighbor Embedding (t-SNE) is a great tool to visualize high-dimensional data in a two-dimensional space by minimizing the Kullback-Leibler divergence. t-SNE visualization of 16 variables for the classification of barefoot and shod groups are shown in Figure 3B.

#### 2.3.2 SVM and Naive Bayes Classifiers

Logistic regression, K nearest neighbor, SVM, Naïve Bayes, decision tree, random forest, and XGBoost were adopted as classifier candidates. In this study, we selected SVM to classify plantar pressure and Gaussian Naïve Bayes to identify foot metrics differences between unshod and shod cohorts based on the performance of 10-fold cross-validation. All variables were standardized during the data preprocessing to a mean of 0 and a standard deviation of 1. Data were split to 70% for training and validation and 30% for testing.

The SVM algorithm constructs a hyperplane that aims to separate between classes in the present dataset by maximizing the margin using support vectors. Feature extraction and selection were performed separately for the SVM algorithm based on the present plantar pressure data. To avoid the limitation of the linear kernel, a Gaussian radial basis kernel was selected for the SVM model.

Regularization combats overfitting by making the model coefficients or weights smaller. More regularization causes lower training accuracy and higher test accuracy (underfitting), and vice versa. A larger C during regularization can lead to overfitting. Specifically, the soft margin parameter of C means the trade-off between margin width and misclassification rate (Fukuchi et al., 2011). A small gamma in the SVM model leads to smoother boundaries, and a larger gamma leads to more complex boundaries. In order to balance the accuracy of the model and avoid overfitting-underfitting problems, hyperparameter tuning using k-fold gride search cross-validation (GridSearchCV) was performed. A C-parameter was chosen as 1 from the range of C: {0.01, 0.1, 1, 10, and 100}, gamma was selected as 0.1 from the range of gamma: {0.0001, 0.001, 0.01, 0.1, and 1}.

To assess the ability of the classifier in predicting categories, 10-fold cross-validation was employed (Fukuchi et al., 2011). Training and validation data were separated into ten subsets to determine the cross-validation performance. Nine subsets were used for training the classifier for each validation, and one subset was used for testing. Accuracy, precision, recall, F1-score, and the Matthews correlation coefficient were employed to evaluate classifiers’ performance.

### 2.4 Statistical Analysis

A Shapiro-Wilk test was performed to examine data normality. The statistical difference between barefoot and shod groups was checked using an independent t-test in Python with the SciPy library. The significance level was set at *p* < 0.05.

## 3 Results

### 3.1 Foot Shape and Plantar Pressure

The foot and heel width are 120.0 ± 11.6 and 62.8 ± 4.8 mm in the barefoot groups, corresponding with 111.1 ± 13.1 mm (*p* < 0.01) and 59.7 ± 3.6 mm (*p* < 0.01) in the shod group (Table 1). Hallux distance and hallux angle present significant differences statistically (*p* < 0.01 and *p* < 0.01).

**TABLE 1:** Participant and foot shape information.

	Barefoot	Shod	t-statistic	*p*
Height (cm)	172.9 ± 5.7	176.0 ± 4.2	−3.78	<0.01*
Mass (kg)	68.7 ± 6.3	71.2 ± 6.1	−2.42	0.02*
BMI (kg/m^2^)	23.0 ± 1.3	22.9 ± 1.4	0.02	0.98
Hallux distance (mm)	25.3 ± 12.1	5.9 ± 6.3	11.84	<0.01*
Hallux angle (°)	0.6 ± 4.4	−8.6 ± 4.7	12.30	<0.01*
Foot length (mm)	259.2 ± 13.0	257.0 ± 11.6	1.08	0.28
Foot width (mm)	120.0 ± 11.6	111.1 ± 13.1	4.37	<0.01*
Heel width (mm)	62.8 ± 4.8	59.7 ± 3.6	4.40	<0.01*
Arch index	0.2 ± 0.02	0.2 ± 0.02	1.22	0.22

Note: * represents *p* < 0.05.

**FIGURE 4:**
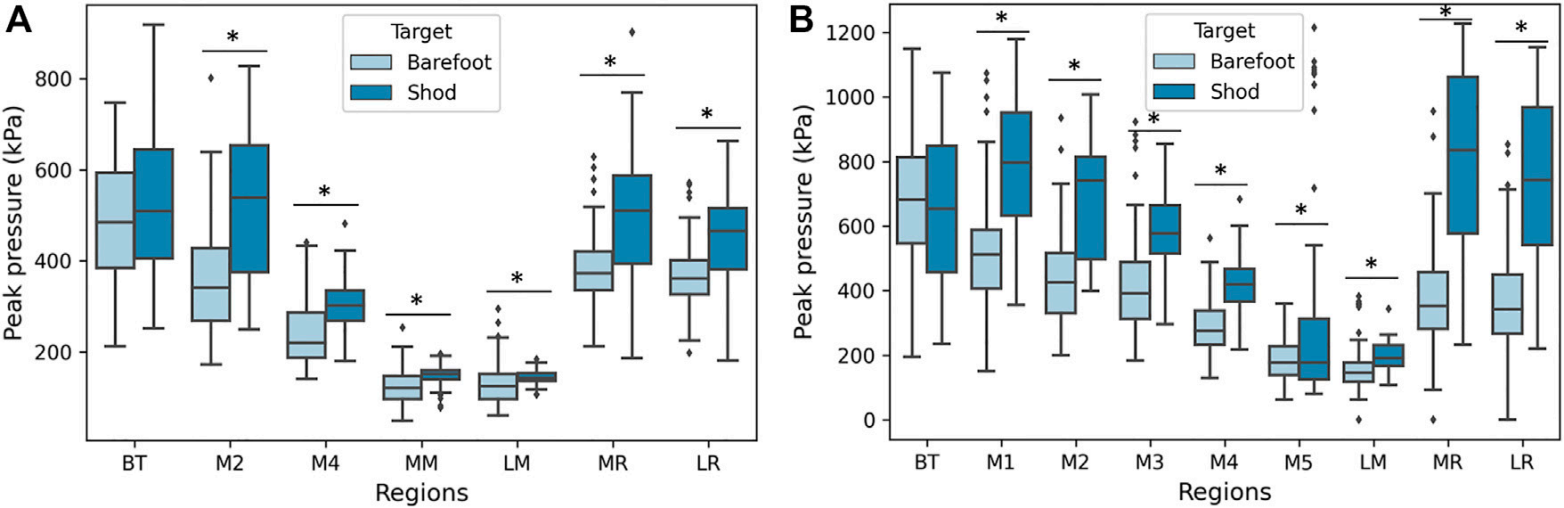
The peak pressure of barefoot and shod cohorts during walking **(A)** and running **(B)**. Note: * represents *p* < 0.05.

The boxplot of plantar pressure for the walking (A) and running (B) between barefoot and shod groups is shown in Figure 4. The independent t-test showed that peak pressure in the barefoot group during walking was decreased significantly in the M2 (33.0%), M4 (21.2%), MM (15.3%), LM (8.8%), MR (24.7%), and LR (19.2%). For the shod group, peak pressure in the M1-M5 during running was significantly higher than the counterparts, increasing 34.8, 37.3, 29.2, 31.7, and 40.1%, respectively. LM, MR and LR also presented the increased peak pressure in the shod group (18.7, 53.3, and 50.8%, respectively).

### 3.2 The Performance of Classifiers

**FIGURE 5:**
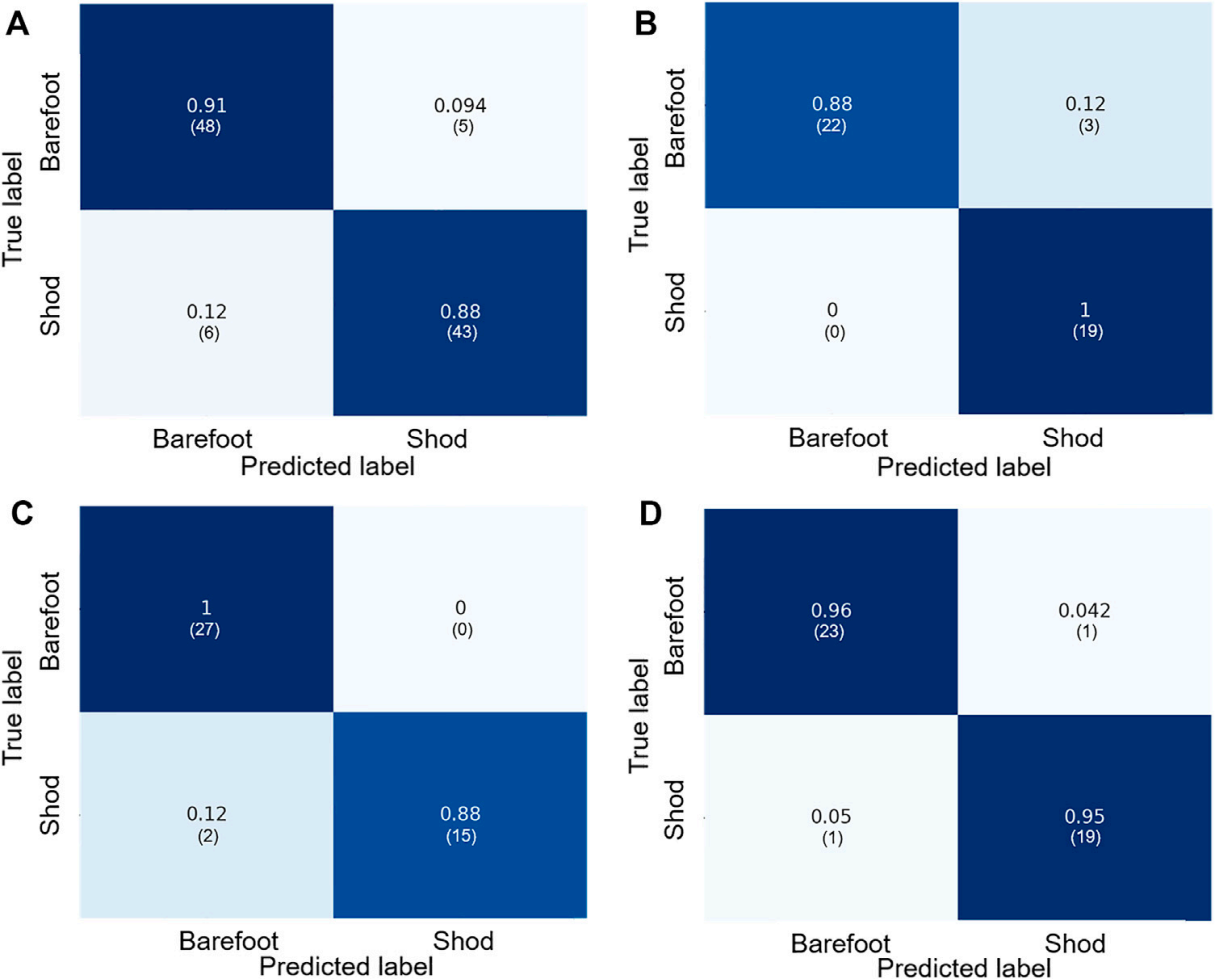
The confusion matrix of training **(A)** and test dataset **(B)** of Naïve Bayes classifier; the confusion Matrix of test dataset for RFE-based SVM model **(C)** and for PCA-based SVM model **(D)**.

Regarding the foot morphology of barefoot and shod populations, the cross-validation accuracy of the Gaussian Naïve Bayes model achieved an accuracy of 89%, while the accuracy of the test dataset achieved was 93% (Table 2). For the resulting RFE-based SVM model, the average 10-fold cross-validation score was 0.98, and the PCA-based SVM model achieved a mean score of 0.96. Both feature extract-based and feature select-based SVM models achieved the test accuracy of 95%. The confusion matrixes of the Gaussian Naïve Bayes and SVM models are presented in Figure 5. The classification report is shown in Table 2.

**TABLE 2:** The classification report of SVM classifiers.

		Number of observations	Cross- validation accuracy	Accuracy	Precision	Recall	F1-score	Matthews correlation coefficient
Naïve Bayes	*Validation dataset*							
	Barefoot	53	0.89		0.89	0.91	0.90	0.78
	Shod	49			0.90	0.88	0.89	
	*Test dataset*							
	Barefoot	25		0.93	1.00	0.88	0.94	0.87
	Shod	19			0.86	1.00	0.93	
SVM	*Validation dataset*							
PCA-based SVM model	Barefoot	54	0.96		0.93	1.00	0.96	0.92
	Shod	48			1.00	0.92	0.96	
RFE-based SVM model	Barefoot	51	0.98		0.98	0.98	0.98	0.96
	Shod	51			0.98	0.98	0.98	
	*Test dataset*							
PCA-based SVM model	Barefoot	24		0.95	0.96	0.96	0.96	0.91
	Shod	20			0.95	0.95	0.95	
RFE-based SVM model	Barefoot	27		0.95	0.93	1.00	0.96	0.91
	Shod	17			1.00	0.88	0.94	

Note: SVM, support vector machine; PCA, principal component analysis; RFE, recursive feature elimination.

## 4 Discussion

Our study proposes a method that enhances routinely used plantar pressure patterns by integrating with foot shape to broaden the classification of shod and barefoot individuals. Plantar pressure is easily measured in gait labs and available in many sports store fitting rooms. Additionally, modern phones are now able to capture foot shape with downloaded apps and our technique is designed to utilise this shape metric to broaden prediction capability. The foot shape of barefoot runners has been clearly documented to be a good predictor in terms of hallux-spacing (Shu et al., 2015). Where our method is advantageous is with amateur runners who are wanting to attempt barefoot running but may not have the appropriate foot shape and plantar pressure profile associated with adapted barefoot runners. Our algorithm will highlight if they share barefoot characteristics and can transition easily, or do not fit the traditional barefoot profile, and should transition with care.

The present study identifies unique foot morphology and plantar pressure patterns between barefoot and shod cohorts via the Naïve Bayes and SVM with the different feature selection and extraction methods. Consistent with our hypothesis, dynamic pressure during locomotion could be utilized to separate and classify the barefoot and shod categories with an accuracy of over 95%. Foot shape also is a critical index for identifying these two groups. Focused on foot shape and pressure to understand and classify them is more accurate and appropriate. Furthermore, barefoot running has got popular in recent years. Some runners may suffer injuries during translating to barefoot running. However, these could not be evaluated or directly measured. Using the machine learning approaches in this study, we could identify people who could and when translated loading patterns during running using foot shape and function measures. Therefore, this method could help identify if gait from a novice barefoot runner translates to habitually barefoot gait.

D’Août et al. (2009) demonstrated that barefoot populations presented relatively longer and wider foot metrics than shod cohorts. Furthermore, wider toe regions are also a significant characteristic for habitual or native barefoot people (Hoffmann, 1905). Shu et al. (2015) illustrated that barefoot runners have a bigger hallux to second toe distance and angle. This study proved that barefoot and shod populations exhibited foot metrics are differentiated and could be separated by the Naïve Bayes classifier. Those differences were mainly in the forefoot (hallux and metatarsal regions) and foot width.

The SVM model in this study was compared with other related studies to evaluate the performance of the classification model (Table 3). Several reports have shown that the SVM classifier is a crucial tool for classification problems in sports medicine and lower limb biomechanics (Xiang et al., 2022; Phinyomark et al., 2015; Christian et al., 2016). In the previous studies, an unsupervised PCA algorithm was commonly used in data preprocessing for discovering the underlying low-dimensional manifolds in high-dimensional datasets (Zdybał et al., 2020) and for data extraction consideration (Wu and Wang, 2008; Taylor et al., 2013; Clermont et al., 2017; Suda et al., 2020). Eleven low-dimensional features were extracted in our study, explaining 90% cumulative variance of the original data. However, intermediate- and higher-order principal components (PCs) may also contain variables to assess the classifiers’ performance (Phinyomark et al., 2015). Furthermore, the extracted topological characteristics may make the findings hard to understand and interpret by the original dataset.

**TABLE 3:** Comparison of the performance of the SVM model in this study with relevant studies.

Study	Subject	Feature	Classifier	Target	Accuracy (%)
Eskofier et al. (2012)	80	Kinematics and kinetics	AdaBoost	barefoot/shod	98.3
Chen et al. (2021)	12	Plantar pressure images	ANN	Walking speeds and durations	94
Terrier, (2020)	36	Center of pressure trajectory	CNN	Footstep recognition	99.9
This study	146	Plantar pressure	SVM	Barefoot/shod	95

Note: ANN, artificial neural network; CNN, convolutional neural network; SVM, support vector machine.

RFE generates the feature coefficients or importance values based on the wrapper-type variable ranking algorithm. The SVM-RFE approach was employed in this study, and variables with the highest relevance got the highest ranking score (Dindorf et al., 2021). It can be used to explore the internal relationship between the original data and the results without extra redundant information. This study found that both PCA and RFE preprocessing methods can build a well-performed classifier with the same accuracy. Future studies shall apply machine learning (i.e., SVM) to identify foot-ankle complexity and gait characteristics with appropriate preprocessing techniques.

Previous studies (Eskofier et al., 2012; Bisele et al., 2017) have identified unique low limb kinematics and kinetics characteristics between barefoot and shod groups during running. Nevertheless, data were collected from the habitually shod cohort. Furthermore, the gait differences were primarily in the plantar loadings following the foot shape (D’Août et al., 2009). This study found the foot shape and plantar pressure differences between habitual shod and barefoot cohorts statistically and from a perspective of machine learning (i.e., SVM and naïve Bayes). Foot functions are linked with morphology. The differential form and function between groups could contribute to understanding the mechanics of running-related injuries in novice barefoot runners because they adopted similar loading patterns but without midsole cushioning from footwear, compared with habitually shod people. Furthermore, this study benefits footwear selection as barefoot cohorts own different forefoot shapes and higher peak pressure during running in the M1-M5 regions.

Given the high running-related injuries in the modern day (Dempster et al., 2021), barefoot running is expected to reduce overall musculoskeletal injury risk as it is the natural way to run biologically (Lieberman, 2012). Barefoot running increases sensory feedback, neuromuscular control, and intrinsic foot muscle strength (Altman and Davis, 2016). Previous studies indicated that barefoot runners with forefoot strike patterns generate smaller collision forces than shod runners presenting rearfoot strike (Lieberman et al., 2010). Also, forefoot strikers exhibited one peak ground reaction force compared with two peak forces in rearfoot strikers during running (Lieberman, 2012). It is known that the habitual barefoot runners are typically loading with a forefoot strike (D’Août et al., 2009; Lieberman et al., 2010). The barefoot population also presents more uniform loading distributions during gait (D’Août et al., 2009). In this study, the plantar pressure during running was significantly decreased in the barefoot group, especially in the rearfoot. However, strike pattern was not part of the screening factor while recruiting the participants, which should be considered when explaining the findings of this study.

However, barefoot or minimalist shoes increase lower extremity instantaneous loading rate, peak heel, and tibia acceleration, compared with wearing cushioned shoes (Sinclair et al., 2016; Agresta et al., 2018). The higher foot and calf injury incidence was presented in the barefoot runners (Altman and Davis, 2016). This study found that the plantar pressure during running showed two typical patterns between the unshod and shod groups. M1 and M5 showed significant differences statistically and are the crucial features that contribute to the good performance of the classifier. It is consistent with previous kinetics findings (Lieberman et al., 2010; Bonacci et al., 2013) that the impact loading in the barefoot running condition was lower than that of the shod group. This finding may be explained by the fact that the participants from the barefoot group have habitually been barefoot since they were born. Therefore, long-term unshod locomotion decreases impact loading and promotes foot strengthening (Miller et al., 2014; Davis et al., 2017).

Furthermore, immediately changing to barefoot or wearing minimal shoes may cause increased injuries among habitually shod runners (Altman and Davis, 2016). This study found that barefoot and shod participants adopted differential loading patterns, regardless of foot strike pattern. Peak pressure is reduced for habitual barefoot runners compared with runners running in cushioned shoes. However, plantar pressure and lower limb loading are the same as shod runners for the novice barefoot runner. Therefore, novice barefoot runners without cushioned footwear protection undertake increased plantar loading than habitual barefoot counterparts during running. Foot pressure difference during running may be a potential factor that causes a high injury rate among novice barefoot runners or runners transitioning to barefoot running, especially in the foot.

Despite the promising findings, this study had the main limitations: the barefoot and shod participants came from different socio-cultural backgrounds. This difference may influence our results. Future studies should evaluate and predict different injury risk factors between barefoot and shod cohorts during gaits and shed light on the injury mechanics for novice barefoot runners. Time and effort are not considered the advantages of this study, as collecting foot pressure and foot anthropomorphic data is more time-consuming than a questionnaire method. However, this study helps to mark the critical biomechanical variables that contributed to classifying barefoot and shod people, particularly foot pressure during running. Classifying barefoot and shod people based on their running using the SVM could accurately identify the gait translation phase from shod running to barefoot running than any other method.

## 5 Conclusion

In summary, the primary advantage of our method is the inclusion of foot shape for broader classification (in addition to plantar pressure). We have trained our model on a population of foot shapes that represent habitually barefoot and shod populations. Classifying feet based on consideration of both shape and plantar pressure is likely to lead to better shoe matching and guidance to practitioners on whether a person’s foot aligns with a barefoot or shod profile. Foot metrics could be identified through the Naïve Bayes algorithm. Furthermore, this study utilized the SVM classifier based on PCA feature extraction and RFE feature selection methods to separate and classify the barefoot and shod populations via walking and running plantar pressure parameters. Forefoot shape could also classify barefoot and unshod populations. Dynamic pressure patterns, especially in the forefoot regions, contribute more to identifying these two cohorts during running.

## Data Availability

The raw data supporting the conclusion of this article will be made available by the authors, without undue reservation.
